# Are there any differences in the provided burn care between men and women? A retrospective study

**DOI:** 10.1186/s41038-018-0125-0

**Published:** 2018-08-13

**Authors:** Laura Pompermaier, Moustafa Elmasry, Islam Abdelrahman, Mats Fredrikson, Folke Sjöberg, Ingrid Steinvall

**Affiliations:** 1 Department of Plastic Surgery, Hand Surgery, and Burns, Linköping University, Linköping, Sweden; 2 Department of Clinical and Experimental Medicine, Linköping University, Linköping, Sweden; 3 Department of Surgery, Plastic Surgery Unit, Suez Canal University, Ismailia, Egypt; 4 Department of Anaesthesia and Intensive Care, Linköping University, Linköping, Sweden; 5 the Burn Centre, Linköping University Hospital, 58185 Linköping, Sweden

**Keywords:** Burn care, Intervention score, Sex, Trauma model, Workload

## Abstract

**Background:**

Disparity between medical treatment for men and women has been recorded worldwide. However, it is difficult to find out if the disparities in both the use of resources and outcome depend entirely on sex-related discrimination. Our aim was to investigate if there are differences in burn treatments between the sexes.

**Methods:**

All patients admitted with burns to Linköping University Hospital during the 16-year period 2000–2015 were included. Interventions were prospectively recorded using the validated Burn SCoring system (BSC). Data were analysed using a multivariable panel regression model adjusted for age, percentage total body surface area (%TBSA), and in-hospital mortality.

**Results:**

A total of 1363 patients were included, who generated a total of 22,301 daily recordings while they were inpatients. Males were 70% (930/1363). Sex was not an independent factor for daily scores after adjustment for age, %TBSA, and mortality in hospital (model *R*^2^=0.60, *p* < 0.001).

**Conclusion:**

We found no evidence of inequity between the sexes in treatments given in our burn centre when we had adjusted for size of burn, age, and mortality. BSC seems to be an appropriate model in which to evaluate sex-related differences in the delivery of treatments.

## Background

In many parts of the world, disparity between the sexes in health care is a reality, with both higher expenditure on health care and a survival advantage for men [1]. In other parts of the world, such differences are less obvious [2–5].

However, it is difficult to find out if the sex-related disparity in medical treatment given depends solely on sex-related discrimination, or if there are possible differences in the underlying medical conditions and their severity [6]. Patients with burns provide an ideal opportunity to study sex-related differences in both interventions and outcomes.

Outcome has been shown by many authors to be related to size of burn and age [7–9], which are strong indicators of severity of illness and underlying medical conditions [7], and the differences in the delivery of medical treatment among sexes can be properly studied using the same variables. Patients with burns are also likely to be cared for in a standard manner and monitored for daily care and treatment.

Sex disparity in the incidence of burns depends on demographic factors, such as age or behaviour, or both, and the causes differ worldwide because of diverse socioeconomic conditions [10]. Scalds and contact burns are the most common thermal injuries in children, regardless of sex [11, 12]; work-related flame burns are most prevalent in adult men; and scalds, inhalation injury, or contact burns that occur at home are the most common causes among adult women internationally [13–16]. Adult women have been reported to be at a disadvantage as far as survival after burns is concerned in both past and recent studies [14, 16–20], whereas mortality in hospital in Sweden is independent of sex [7, 21].

It has recently been reported that the amount of resources used for the care of patients in Swedish general intensive care has been to the benefit of the men [22], and for this reason, it would be interesting to analyse whether the requirement or delivery of treatment to burned patients is equal between the sexes.

The therapeutic Burn SCoring system (BSC) has been designed in 1992 at our centre to assess the medical interventions made on each patient, as well as to calculate the cost of each patient’s treatment [23]. It has developed principally from the Nursing Care Recording system, which is an intervention scoring system built for general intensive care, including burn-specific items, such as burn excisions, skin grafting, and change of dressing. To validate the BSC, the scores obtained from the same patient with the BSC were compared with those obtained with the validated Therapeutic Intervention Scoring System (TISS), showing good agreement in the assessment of level of care. Furthermore, the BSC has been described in a recent publication about the use of resources in relation to outcome (mortality) [24].

**Table 1:** Details of the patients admitted with burns during 2000–2015

	Female patients		Male patients		*p* value
No. of patients	403		960		
Age (years)	29.6	1.2–76.2	33.9	1.3–71.4	0.10
TBSA burned (%)	7.0	1.0–27.0	6.4	1.0–32.8	0.37
Hospital stay (days)	7.0	2.0–39.0	8.0	2.0–35.0	0.52
No. of deaths	25 (6)		45 (5)		0.25
No. who required mechanical ventilation	83 (21)		195 (20)		0.91
BSC categories					
Surveillance	4.0	0.0–57.0	3.0	0.0–57.0	0.23
Respiration	1.0	0.0–44.0	0.0	0.0–40.5	0.54
Circulation	0.0	0.0–9.0	0.0	0.0–7.5	0.22
Wound care	11.0	1.0–66.0	11.0	1.0–60.0	0.65
Mobilisation	9.0	2.0–66.0	8.0	2.0–62.0	0.41
Laboratory tests	1.0	0.0–26.0	2.0	0.0–28.0	0.42
Infusions	4.0	0.0–55.0	3.0	0.0–62.5	0.65
Operation	4.0	0.0–24.0	4.0	0.0–33.0	0.73
Total BSC points	36.0	7.0–350.0	34.0	7.0–345.0	0.37

*TBSA* total body surface area, *BSC* Burn SCoring system

Data are presented as median and 10th–90th centiles or number and percentage

The aim of the present study was to investigate potential differences between the treatment of men and women in intensive care as well as in the general ward. We used total daily recordings of the BSC (for each patient) for analysis, and adjusted for age, percentage total body surface area (%TBSA), and in-hospital mortality.

## Methods

All patients admitted to Linköping University Hospital Burn Centre since 1 January 2000 and discharged before the 10 November 2015 were included. We analysed the data that were recorded in the prospectively maintained burn registry [23]. The study was approved by the Regional Ethics Review Board in Linköping (No.2013/341-31).

Patients were treated with early excision and grafting [23], and revision of the wound every second day. The patients with the most severe burns and those who required intensive care for other reasons were treated with standard ventilation [25, 26], fluid management [27], and early enteral nutrition.

Those with minor burns had early tangential excision, and the burn was covered with meshed split-thickness skin grafts. Major burns were treated by staged excisions and covered with xenografts to allow clear demarcation of the wound bed, followed by a later autografting [28–30]. All the procedures, whether operations or dressings under sedation, were done within the burn centre’s operating rooms and handled by the staff of the unit, except for intraoperative duties such as anaesthesia and scrubbing.

General variables measured and included in the study are as follows: %TBSA, cause of injury, age, sex, duration of hospital stay, and survival. Mortality was defined as death from any cause during admission to the burn centre. The BSC covers the following categories of care: surveillance, respiration, circulation, wound care, mobilisation, laboratory tests, infusions, and operations [23]. Each category is given a score from 0 to 4 depending on the level of care, from the less challenging (BSC = 0) to the most (BSC = 4). An exception is the scoring of the operation, which is calculated based on the operating time and 1 h equals a score of 2, 2 h a score of 4, etc. In practice, the more points are scored, the more has been the work done.

Daily BSC refers to the score recorded for each patient every 24 h, and total BSC refers to the sum of the daily BSC points for each patient.

We defined patients who required intensive care as those who needed mechanical ventilation during their stay at the burn centre, and days in intensive care as those days when patients in the unit required mechanical ventilation.

### Analysis of data and statistics

Data were analysed with STATA (STATA v12.0, Stata Corp. LP College Station, TX, USA) and presented as median (10–90 centiles) unless otherwise stated. The significance of differences in characteristics was assessed with the Mann-Whitney *U* test and the chi-square test unless otherwise stated. Simple linear regression was used for the scattergrams. As the hypothesis was that there was no difference between the treatment given to men and women, we did a non-inferiority analysis using 1 BSC point/day as the limit (delta). A multivariable regression for panel data (panel variable by patient) was used for the analysis of the association between sex and daily BSC points. A subgroup analysis was done for the patients in intensive care using the same regression model. Probabilities of less than 0.05 were accepted as significant.

## Results

The data from 1363 patients were analysed based on 22,301 daily inpatient recordings of BSC. The median age was 33 years (10th–90th centiles, 1–72), %TBSA was 7% (10th–90th centiles, 1–31), 960 of the 1363 were male (70%), and crude mortality was 5.1%. There were no differences between the groups of male and female patients regarding total BSC points/patient, BSC points by the eight BSC categories, duration of hospital stay, mortality, size of the burn, or age (Table 1).

**Fig. 1:**
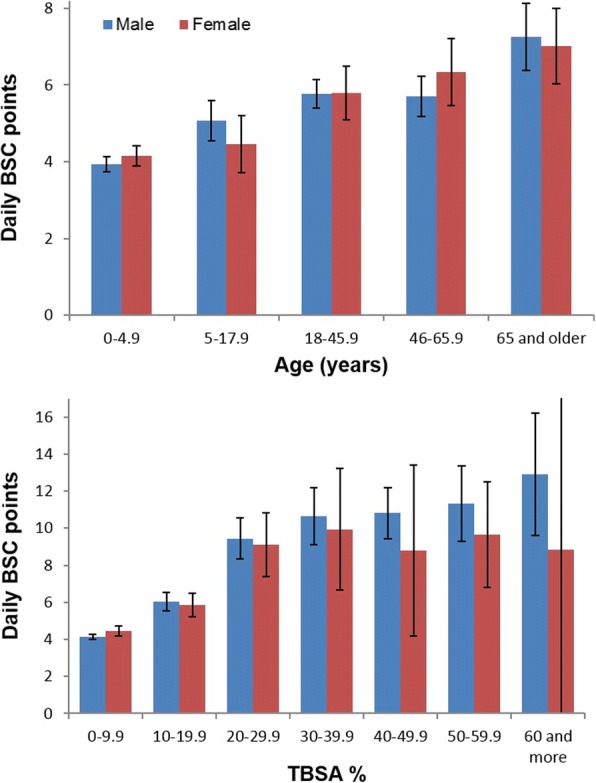
Mean (95% confidence interval) daily Burn SCoring system (BSC) points among the patients who survived. Upper figure: subgroups by age, lower figure: by %TBSA groups.

Figure 1 shows the mean daily BSC points among the patients who survived, according to their ages and %TBSA groups. Significant differences (unadjusted) between male and female patients were found in one age group (46–65.9 years, *p* = 0.046) and one %TBSA group (0–0.9%, *p* = 0.02).

The unadjusted mean difference was 0.8 BSC points/day higher in the male group (mean (SD) 8.9 (7.5)) compared with the female group (mean (SD) 8.1 (6.6)) when we analysed the 22,301 daily recordings (*t* test, *p* < 0.001). Non-inferiority analysis showed that if there was no difference in daily BSC between male and female patients, then 1778 recordings would be required to be 90% sure that the upper limit of a one-sided 95% confidence interval (CI) (or equivalently a 90% two-sided CI) will be below the non-inferiority limit of 1 (SD of outcome 7.2). When using the actual mean difference 0.8, the required number was 2776 recordings, which is one eighth of the 22,301 that we used in the regression model.

The multivariable regression model showed that sex was not an independent factor for daily BSC points (Table 2), which increased with the size of the burn, in older patients and for those that died during their admission.

**Table 2:** The association between sex and daily Burn SCoring system points, adjusted for total body surface area (TBSA) burned, age, and deaths

	Coefficient	SE	*p* value	95% CI
TBSA burned (%)				
0–9.9				
10–19.9	1.89	0.14	< 0.001	1.62 to 2.16
20–29.9	4.98	0.17	< 0.001	4.65 to 5.31
30–39.9	6.15	0.20	< 0.001	5.75 to 6.55
40–49.9	6.59	0.25	< 0.001	6.10 to 7.09
50–59.9	6.36	0.26	< 0.001	5.85 to 6.87
60 and more	8.06	0.28	< 0.001	7.52 to 8.60
Age (years)				
0–4.9				
5–17.9	− 0.38	0.23	0.10	− 0.84 to 0.07
18–45.9	1.21	0.18	< 0.001	0.87 to 1.56
46–65.9	2.00	0.18	< 0.001	1.65 to 2.35
65 and older	3.04	0.19	< 0.001	2.67 to 3.42
Sex (male)	0.12	0.12	0.30	− 0.11 to 0.34
Mortality	6.44	0.25	< 0.001	5.95 to 6.93
Constant	3.06	0.17	< 0.001	2.74 to 3.39

*SE* standard error, *CI* confidence interval

Multivariable regression for panel data, model (between) *R*^2^=0.60, *p* < 0.001. Patients *n* = 1363, daily recordings *n* = 22,301

We also tested to add the type of burn (hot object, chemicals, flame, electricity, and others) in our regression model but it did not change the result (coefficient for sex (male)=0.09, *p* = 0.41). The subgroup analysis of the 278 patients who required intensive care showed similar results, in that there was no difference in the number of daily BSC points between male and female patients during the period of intensive care (Table 3).

**Table 3:** The association between sex and daily Burn SCoring system (BSC) points during the period of intensive care (treatment with mechanical ventilation)

	Coefficient	SE	*p* value	95% CI
TBSA burned (%)				
0–9.9				
10–19.9	1.55	0.68	0.02	0.21 to 2.89
20–29.9	2.25	0.62	< 0.001	1.04 to 3.45
30–39.9	2.83	0.65	< 0.001	1.56 to 4.10
40–49.9	3.30	0.71	< 0.001	1.92 to 4.68
50–59.9	2.88	0.74	< 0.001	1.44 to 4.32
60 and more	4.11	0.71	< 0.001	2.72 to 5.49
Age (years)				
0–4.9				
5–17.9	2.93	1.17	0.01	0.65 to 5.22
18–45.9	3.71	0.97	< 0.001	1.81 to 5.61
46–65.9	3.51	0.96	< 0.001	1.62 to 5.40
65 years and over	2.70	1.00	0.007	0.74 to 4.66
Sex (male)	− 0.07	0.33	0.83	− 0.72 to 0.58
Mortality	2.13	0.41	< 0.001	1.33 to 2.93
Constant	12.89	1.08	< 0.001	10.76 to 15.01

*SE* standard error, *CI* confidence interval, *TBSA* total body surface area

Multivariable regression for panel data, model (between) *R*^2^=0.16, *p* < 0.001. Patients *n* = 278, daily recordings *n* = 4427

## Discussion

We have analysed 22,301 daily intervention score recordings using a multivariable regression model to adjust for size of burn, age, and mortality, and we have found no evidence of inequity among sexes in treatments given in our burn centre.

Measurements of the workload in general intensive care units have been used to measure the quality of care [31–33]. Systems designed to “score” different treatments of burns are few, and those currently available tend to depict local policies of care [34, 35] and are designed for use in general intensive care units [16, 36, 37]. In particular, management of the wound is an activity specific to the care of burns that is vital, and must be incorporated in the measurement of workload [37, 38].

The BSC was developed to remedy this deficiency, and we have used it to analyse the delivery of care. Assuming that the causes of burns differ between sexes [39], we investigated whether different types of burns (flame, scald, chemical, and electrical) influence treatment at the burn centre and we found that they did not.

However, patients with burns provide an excellent model for the study of sex-related differences in both interventions and outcomes, as the group is homogeneous with a common cause for admission (the burn), the time of the injury is known, the medical care is relatively standard, and 97% of the mortality may be explained by the two variables %TBSA and age [40]. Furthermore, age is a good surrogate marker for coexisting disease [7] and %TBSA for burn severity and can be quantified [17, 29].

Several international studies have reported that adult men have a survival advantage after burns [14–19], which is in contrast to our finding that the sex of the patient does not seem to be an independent factor for mortality among patients with burns [7, 21]. It is not possible to deduce if this is caused by the underlying severity of injury or by differences in treatment, as the studies mentioned above did not include treatments in their analysis.

In a recent Swedish study, male patients have been shown to receive better care than women in general intensive care units. We could not confirm this inequity among intensive care patients with burns in our subgroup analysis, despite Swedish general intensive care units and those specifically for patients with burns are thought to be comparable and their routines are similar. Both are managed by intensive care physicians who work in both the general intensive care unit and the intensive care unit at the burn centre. The intervention scoring system currently in use in general intensive care units [22] and the BSC [23] are both modified versions of the original Nursing Care Recording system [41] and are consequently regarded as equivalent. These conflicting findings could possibly be explained by the use of different methods to adjust for the severity of illness. Samuelsson et al. [22] used the Simplified Acute Physiology Score III (SAPS III) to adjust for severity of illness, whereas we used the %TBSA, which is an excellent measure of the severity of a burn [9, 40], being more sensitive and having a greater degree of accuracy. It would, however, have been interesting to test if we would have found differences in provided burn care between men and women, using the SAPS III as this possibly would have resulted in an insufficient adjustment. Unfortunately, SAPS III is not recorded in our database.

### Limitations of the study

Firstly, although the BSC is a detailed scoring system with its seven care categories, it cannot be considered to be complete. It may be that it is not detailed enough to detect possible differences in care, but it has been found to correlate closely with the TISS, which is a detailed system with 36 different scores, and so may still be claimed to be a valid instrument. Secondly, this study was a single-centre investigation made in a country with a limited and quite homogeneous population, and this may reduce the generalizability of its conclusions. However, patients with burns admitted to our centre comprise all patients with burns without exclusion for age, sex, severity of burn, or cause of injury, and some global similarities, such as the decreases in the incidence of burns; the severity of injury, mortality, and the duration of stay [42], give value to our results. Yet, using the same intervention scoring system in other burn centres with outcome figures different than ours would be interesting to validate this instrument. Thirdly, although the treatments at the burn centre have been repeatedly investigated and the results published, and there have been improvements in outcome during the duration of this study [24], there may also be a time-related effect. From a scientific point of view, however, it is unlikely that this would affect the sex-related treatments being studied. Fourthly, large volumes of daily recordings made over time always carry a risk of the results being faulty and inconsistent. For this particular study, we claim that this is less likely as a well-known score has been used, and the categories would be difficult to judge other than objectively.

## Conclusion

This is the first study to our knowledge that has investigated whether there are sex-related differences in the treatments given over a long period of time to patients with burns. It is the second from this unit in which we have studied sex-related differences in outcome in a strictly adjusted model. In line with the previous publication, in which a similar mortality between the sexes was recorded, we have been unable to find data that indicate inequities between the sexes in the treatments provided. The model of burns, in which detailed adjustments can be made for severity of illness and pre-existing medical conditions, seems to be suitable for the evaluation of sex-related differences in the delivery of medical care.
